# Effects of tai chi based on information and communication technology for patients with mild cognitive impairment on cognitive and physical function: a systematic review and meta-analysis

**DOI:** 10.3389/fpubh.2024.1495645

**Published:** 2025-01-07

**Authors:** Yezi Li, Qingjie Wang, Yuanyuan Ren, Xiaokun Mao

**Affiliations:** ^1^School of Physical Education and Sports, Soochow University, Suzhou, Jiangsu, China; ^2^Suzhou City University, Suzhou, Jiangsu, China

**Keywords:** tai chi, mild cognitive impairment, cognition, physical function, older adults

## Abstract

**Objective:**

This study evaluated the effectiveness of tai chi, enhanced by communication technologies, in improving cognitive and physical functioning in patients with mild cognitive impairment, and to compare these effects with traditional tai chi.

**Methods:**

A systematic search across four academic databases identified 16 studies with 1,877 participants. Data were expressed as weighted or standardized mean differences with 95% confidence intervals.

**Results:**

A meta-analysis revealed significant improvements in Mini-Mental State Examination scores and Timed Up and Go results in patients with mild cognitive impairment following tai chi intervention. Subgroup analysis indicated that both communication technology-based tai chi and traditional tai chi produced varying improvements in cognitive and physical function.

**Conclusion:**

This study confirms the importance of tai chi for cognitive and physical functioning in patients with mild cognitive impairment. Compared with traditional tai chi, communication technology-based tai chi showed greater benefits in promoting rehabilitation. The effective and feasible interventions could improve the physical health of many older adult patients, these findings provide valuable insights and decision-making guidance for clinical practice and public health with older patients with mild cognitive impairment.

**Systematic review registration:**

PROSPERO, CRD42023449711, available from: https://www.crd.york.ac.uk/prospero/display_record.php?ID=CRD42023449711.

## 1 Introduction

According to the World Health Organization, dementia has become the leading mental and neurological disorder among individuals aged 60 years, with the number of affected individuals increasing annually. Mild cognitive impairment (MCI), which often precedes dementia, is of particular concern and affects approximately 16% of the older adult population (1). Individuals with MCI are at high risk of progressing to dementia unless appropriate preventive and therapeutic measures are implemented.

Tai chi has been shown to effectively mitigate cognitive decline in older adult patients by promoting the integration of body and mind (2–8). Traditional tai chi, delivered through on-site instruction and exercises, has commonly been used to improve cognitive function in older adults. However, recent studies have explored the use of information and communication technologies (ICT), such as virtual reality, video games, and online platforms, to facilitate tai chi exercises for older adult individuals, particularly those with cognitive impairment. ICT interventions are non-pharmacological approaches that utilize digital devices such as computers, touchscreens, and virtual reality. For example, during the COVID-19 pandemic, a study utilized video conferencing to guide tai chi exercises, preventing offline gatherings. This ICT-based intervention effectively alleviated anxiety and behavioral symptoms in individuals with MCI (9). Other studies suggest that combining ICT with physical exercise and physiotherapy can enhance patients’ ability to perform daily activities (10, 11). Additionally, ICT-based tai chi exercises may be beneficial in implementing exercise interventions for older adults (9, 12, 13). As video game technologies such as virtual reality continue to gain popularity, the development of rehabilitation training programs has increased. However, few studies have compared the effects of ICT-based tai chi to traditional tai chi on older adult individuals with MCI.

Therefore, this study aimed to further investigate the effect of ICT-based tai chi on the cognitive and physical functions of individuals with MCI, comparing its effectiveness with that of traditional tai chi. The goal is to optimize tai chi rehabilitation programs for individuals with MCI and provide new evidence and recommendations for clinical treatments.

## 2 Methods

This systematic review and meta-analysis adhered to the Preferred Reporting Items for Systematic Reviews and Meta-Analyses (PRISMA) guidelines (14). The review protocol was registered with PROSPERO (ID = CRD42023449711).

A systematic literature search was conducted up to November 7, 2024, using the terms “tai chi,” “online tai chi,” “VR-based tai chi,” “exergaming-based tai chi,” “Mild Cognitive Impairment,” “Cognitive Function,” and “Physical Function.” Databases searched included PubMed, Web of Science, Elsevier, Cochrane, and others, with results limited to English-language publications.

Inclusion criteria were as follows: (1) patients with MCI, defined by scores <25 on the Mini-Mental State Examination or ≤ 0.5 on the Clinical Dementia Rating; (2) randomized controlled trials and quasi-experimental studies, where the experimental group received tai chi intervention (traditional, online, VR-based, or exergaming-based), and the control group had no intervention, routine intervention, or daily physical activity; and (3) studies reporting one or more outcomes related to cognitive function, physical function, balance, or falls.

The exclusion criteria were as follows: (1) Studies involving individuals with Alzheimer’s disease, normal cognitive function, or comorbidities such as cancer, degenerative neurological disorders, or major depression; (2) Intervention programs that included confounding factors, such as cognitive training, vitamin supplements, or medications, alongside exercise; (3) Articles classified as reviews, systematic evaluations, or experimental studies that did not include tai chi interventions; (4) Experimental studies failing to meet the specified outcome metrics; and (5) Duplicate experimental studies not meeting the outcome criteria.

All studies were initially imported into the literature management system. After duplicates were removed, two reviewers independently screened studies based on titles and abstracts. The remaining articles were reviewed in detail and assessed against the inclusion and exclusion criteria. Disagreements were resolved through discussions with a third reviewer.

Two reviewers independently extracted data from the selected articles, which included basic study information, participant demographics, methodology, frequency and duration of tai chi interventions, control conditions, and outcomes. Methodological quality was assessed using the revised PEDro scale, with 11 criteria scored on a 2-point scale (1 for compliant, 0 for non-compliant). The quality scoring was performed independently by two reviewers, and disagreements were addressed through discussions with a third reviewer. The assessed domains included randomization procedures, intervention fidelity, handling of missing outcome data, outcome measurement, and outcome reporting. Publication bias was evaluated using funnel plots.

Data analysis was conducted using RevMan 5.3 software, adhering to PRISMA guidelines. As the study data consisted of continuous variables, results were presented as weighted or standardized mean differences with 95% confidence intervals, using a significance level of *α* = 0.05. Heterogeneity was assessed with the *I^2^* test, and sensitivity analysis was performed if significant heterogeneity was detected. If heterogeneity remained without notable changes, a random-effects model was applied. When the source of heterogeneity could not be identified, qualitative analysis was used. Publication bias was assessed with Stata 16.0 software, and its impact on the overall effect was evaluated using the cut-and-patch method.

## 3 Results

### 3.1 Literature search results and basic characteristics

**Figure 1:**
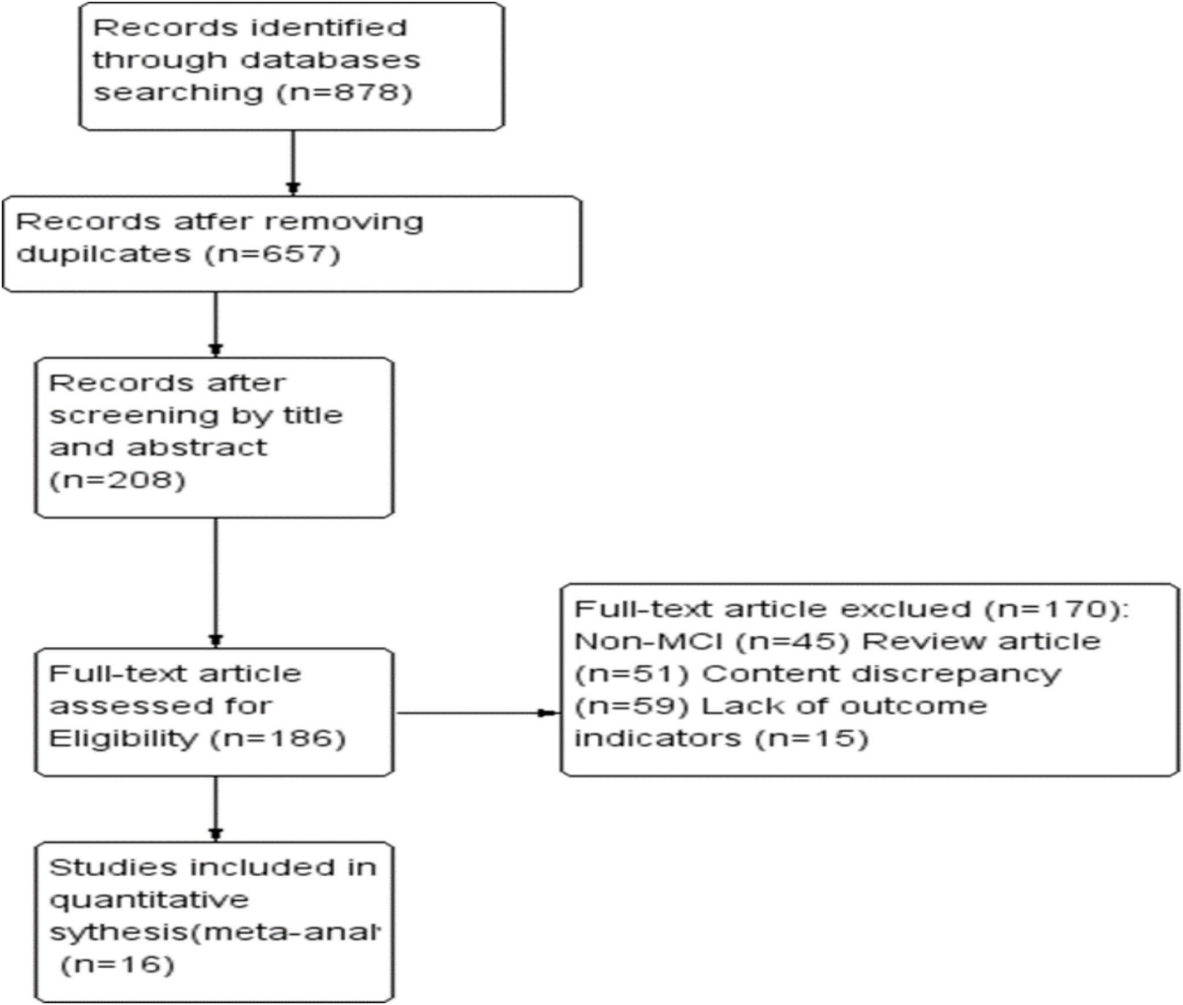
Flow diagram of included and excluded studies.

Initially, 878 articles were identified. After a preliminary screening of titles and abstracts, followed by manual searches of selected works, 16 full articles were ultimately included in this study (Figure 1).

Sixteen studies conducted between 2010 and 2024, with a total of 1,877 participants, were included in the analysis (Table 1). Three of these studies used videoconferencing for teaching and exercise, two employed virtual reality or video games for tai chi interventions, and the remaining 11 utilized traditional methods. The studies were primarily conducted in Asia and North America, with 10 studies in China, two in Thailand, three in the United States, and one in Brazil. Intervention durations varied from 20 to 90 min, and cycles ranged from 7 weeks to 1 year. Several studies (7, 15–20) reported outcomes for different intervention cycles, with frequencies ranging from 1 to 5 times per week.

**Table 1:** Characteristics of included studies.

Studies	Region	Mean Age (Mean ± SD)	Sample Size (Male/Female)	Intervention	Control	Outcomes
Hsieh et al., 2018 (15)	Taiwan	IG: 76.4 ± 7.6 CG: 80 ± 7.5	IG: 31 (7/24) CG: 29 (10/19)	VR-based TC (ICTTC)	Usual daily physical activities	Physical function: 30s STS, TUG, GS.
Hwang et al., 2023 (41)	Taiwan	IG: - CG: -	IG: 35 (−/−) CG: 49 (−/−)	Traditional TC	Health education	Physical function: TUG.
Jiayuan et al., 2022 (7)	Harbin	IG: 71.7 ± 3.9 CG: 70.8 ± 4.2	IG: 29 (11/18) CG: 30 (13/17)	Traditional TC	Mindfulness intervention	Cognitive function: MMSE; Physical function: 30s STS, TUG.
Kasai et al., 2010 (42)	Brazil	IG: 73.54 CG: 74.54	IG: 13 (0/13) CG: 13 (0/13)	Traditional TC	None	Cognitive function: DSF, DSB.
Lam et al., 2011 (43)	Hong Kong	IG: 77.2 ± 6.3 CG: 78.3 ± 6.6	IG: 171 (46/125) CG: 218 (46/172)	Traditional TC	Muscle stretching exercise	Cognitive function: MMSE, DSF, DSB, TMT-A, TMT-B, delayed recall, VFT. Physical function: BBS.
Lam et al., 2012 (44)	Hong Kong	IG: 77.2 ± 6.3 CG: 78.3 ± 6.6	IG: 171 (46/125) CG: 218 (46/172)	Traditional TC	Muscle stretching exercise	Cognitive function: MMSE, DSF, DSB, TMT-A, TMT-B, delayed recall, VFT. Physical function: BBS.
Li et al., 2014 (45)	USA	IG: 75 ± 11 CG: 77 ± 10	IG: 22 (7/15) CG: 24 (7/17)	Traditional TC	None	Cognitive function: MMSE. Physical function: TUG, GS.
Li et al., 2021 (29)	Hong Kong	IG: 76.13 ± 6.2 CG: 76.2 ± 6.3	IG: 15 (6/9) CG: 15 (3/12)	Online TC (ICTTC)	Stretching	Physical function: 30s STS, TUG
Li et al., 2022 (8)	USA	IG: 74.5 ± 5.6 CG: 74.9 ± 6.3	IG: 22 (14/8) CG: 24 (9/15)	Online TC (ICTTC)	Stretching	Cognitive function: MoCA, DSF, DSB, TMT-B, VFT.
Li et al., 2023 (16)	USA	IG: 75.9 ± 5.1 CG: 76.0 ± 6.1	IG: 107 (41/66) CG: 106 (35/71)	Online TC (ICTTC)	Stretching	Cognitive function: DSF, DSB, TMT-B, VFT, Physical function: TUG, 30s STS
Liu et al., 2022 (17)	Taiwan	EXER-TC: 74.6 ± 6.1 TC: 73.2 ± 6.3 CG: 73.4 ± 6.5	EXER-TC: 16 (4/12) TC: 17 (5/12) CG: 17 (6/11)	EXER-TC (ICTTC)/Traditional TC	None	Cognitive function: MoCA, TMT-A, TMT-B, delayed recall.
Siu and Lee. 2018 (46)	Hong Kong	IG: - CG: -	IG: 80 (22/58) CG: 80 (20/60)	Traditional TC	Recreational activity	Cognitive function: MMSE.
Sun et al., 2015 (47)	Jilin	IG: 68.3 ± 5.9 CG: 70.1 ± 5.7	IG: 72 (14/58) CG: 66 (20/46)	Traditional TC	Non-athletic activities	Cognitive function: MMSE. Physical function: GS
Sungkarat et al., 2017 (25)	Thailand	IG: 68.3 ± 6.7 CG: 67.5 ± 7.3	IG: 33 (2/31) CG: 33 (7/26)	Traditional TC	None	Cognitive function: TMT B-A, delayed recall.
Sungkarat et al., 2018 (26)	Thailand	IG: 68.3 ± 6.7 CG: 67.5 ± 7.3	IG: 33 (2/31) CG: 33 (7/26)	Traditional TC	None	Cognitive function: TMT B-A, delayed recall.
Yu et al., 2022 (18)	Hong Kong	TC: 67.3 ± 4.2 EX: 67.3 ± 4.2 CG: 67.2 ± 6.8	TC: 10 (3/7) EX: 10 (3/7) CG: 12 (2/10)	Traditional TC	Conventional exercise/None	Cognitive function: MoCA, TMT-A, TMT-B, TMT B-A, VFT. Physical function: 30s STS.

IG, intervention group; CG, control group; TC, tai chi; ICTTC, information and communication technologies-based Tai Chi; EXER-TC, exergaming-based tai chi group; 30s-STS, 30s sit to stand test; TUG, timed up to go; GS, gait speed; MMSE, mini-mental state examination; MoCA, montreal cognitive assessment; DSF, digital span forward; DSB, digital span backward; TMT-A, trail making test A; TMT-B, trail making test B; TMT B-A, trail making test B-A; VFT, verbal fluency test; BBS, berg balance scale.

### 3.2 Literature quality and risk of publication bias

Table 2 displays the results of the literature quality assessment based on the PEDro scale, with scores categorized as follows: 0–4 for low-quality studies, 5–7 for medium-quality studies, and 8–10 for high-quality studies.

**Table 2:** Evaluation results of PEDro scale of the included studies.

Studies	PEDro scale											
	1	2	3	4	5	6	7	8	9	10	11	Totals
Hsieh et al., 2018 (15)	Yes	0	0	1	0	0	0	1	1	1	1	5
Hwang et al., 2023 (41)	Yes	1	1	1	0	0	0	1	1	1	1	7
Jiayuan et al., 2022 (7)	Yes	1	1	1	0	0	0	1	1	1	1	7
Kasai et al., 2010 (42)	Yes	0	0	1	0	0	0	1	1	1	1	5
Lam et al., 2011 (43)	Yes	1	1	1	0	0	1	1	1	1	1	8
Lam et al., 2012 (44)	Yes	1	1	1	0	0	1	1	1	1	1	8
Li et al., 2014 (45)	Yes	0	0	1	0	0	0	1	1	1	1	5
Li et al., 2021 (29)	Yes	1	1	1	0	0	1	1	1	1	1	8
Li et al., 2022 (8)	Yes	1	1	1	0	0	1	1	1	1	1	8
Li et al., 2023 (16)	Yes	1	1	1	0	0	1	1	1	1	1	8
Liu et al., 2022 (17)	Yes	1	1	1	0	0	1	1	1	1	1	8
Siu and Lee, 2018 (46)	Yes	0	0	1	0	0	0	1	1	1	1	5
Sun et al., 2015 (47)	Yes	1	0	1	0	0	0	1	1	1	1	6
Sungkarat et al., 2017 (25)	Yes	1	1	1	0	0	1	1	1	1	1	8
Sungkarat et al., 2018 (26)	Yes	1	1	1	0	0	1	1	1	1	1	8
Yu et al., 2022 (18)	Yes	1	1	1	0	0	1	1	1	1	1	8

**Figure 2:**
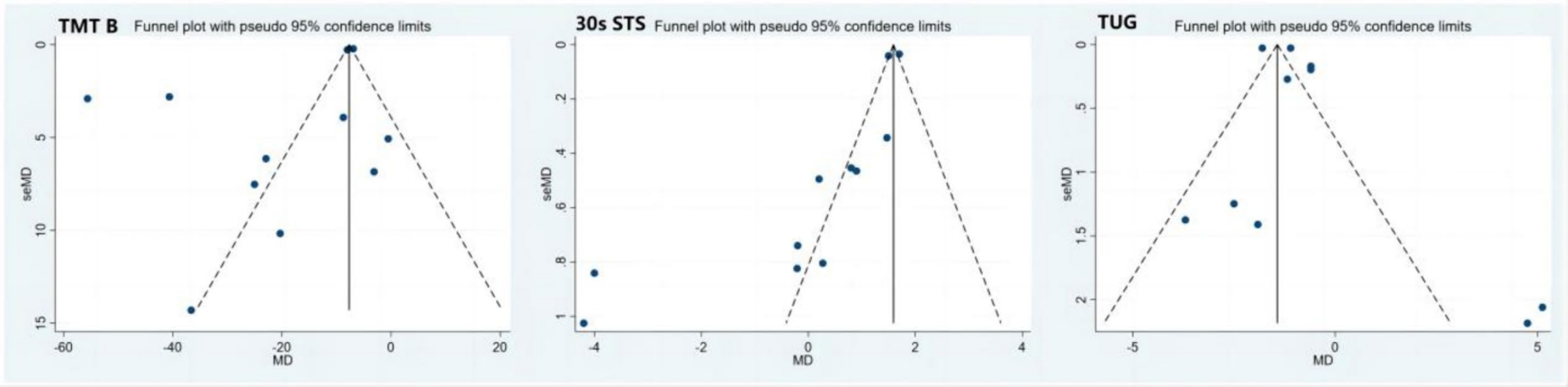
Funnel plots of 30 s STS, TUG and TMT B. 30 s-STS, 30 s sit to stand test; TUG, timed up to go; TMT-B, trail making test B.

**Figure 3:**
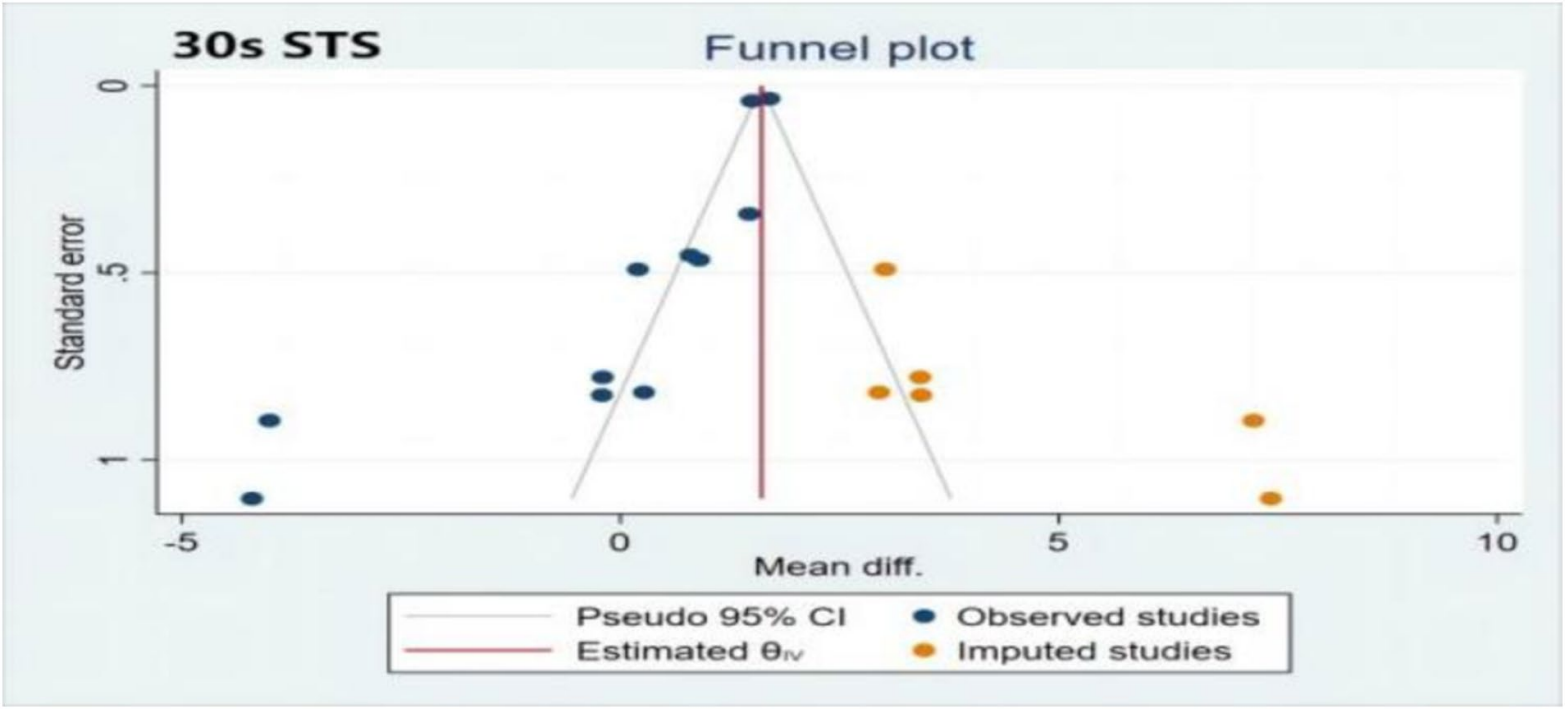
Trim and fill funnel plot of 30s STS. 30 s-STS, 30 s sit to stand test.

Figure 2 shows the results of the publication bias assessment for indicators of the 30-s STS, TUG, and TMT B. No significant publication bias was found for the timed up-and-go (TUG) and trail making test B (TMT B). However, there was significant publication bias for the 30-s STS (*t* = −3.95; *p* = 0.003; 95% CI: −4.89 to −1.32). To further assess the impact of publication bias, the cut-and-patch method was applied. Figure 3 indicates that publication bias had minimal effect on the 30-s STS results (MD = 1.61; 95% CI: 1.56 to 1.66), demonstrating the robustness of the findings.

### 3.3 Cognitive function

**Figure 4:**
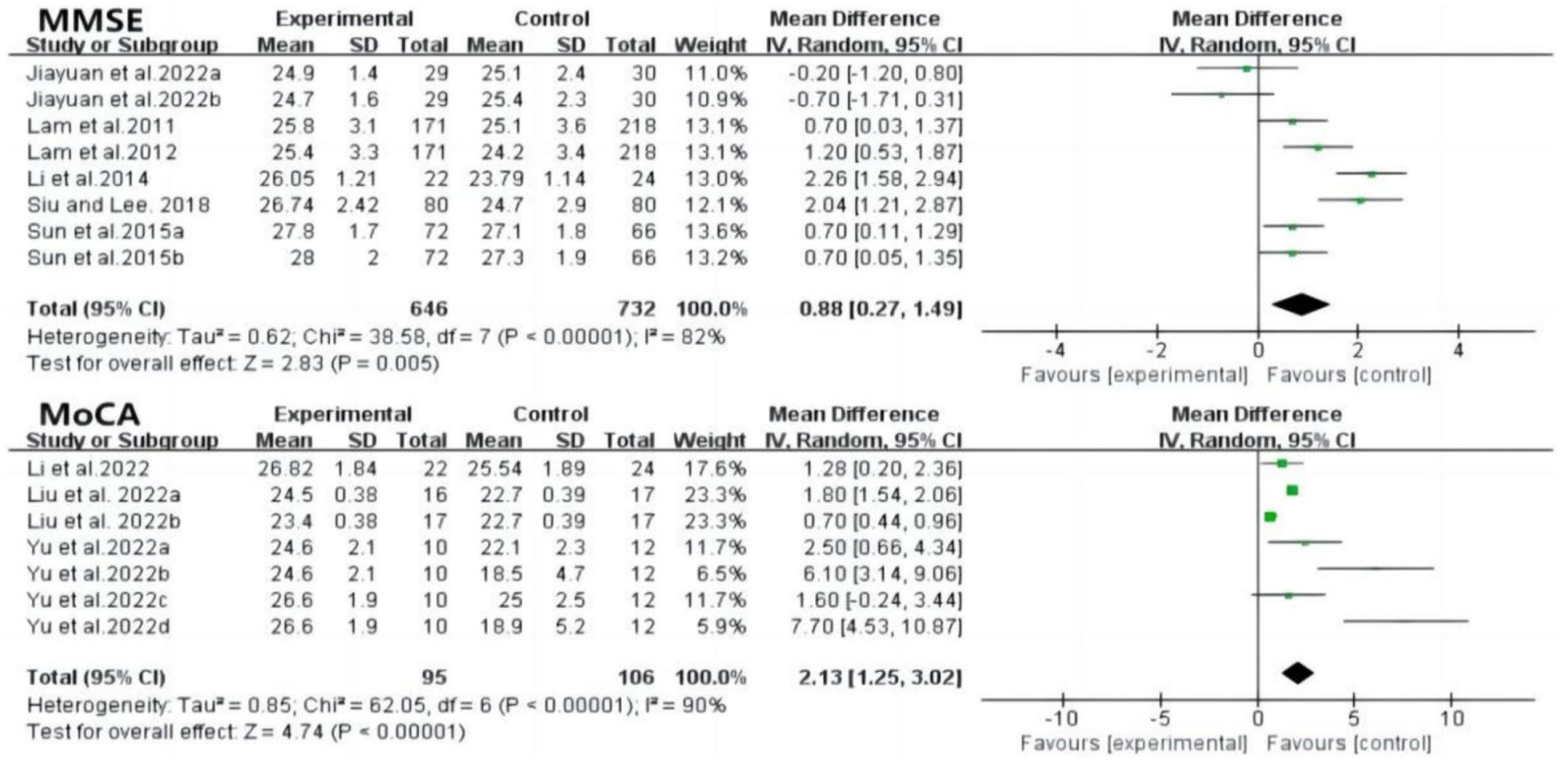
Forest plot of MMSE and MoCA after tai chi intervention. MMSE, mini-mental state examination; MoCA, montreal cognitive assessment.

Figure 4 also depicts the effects of tai chi on global cognitive function. Eight studies reported mini-mental state examination (MMSE) outcomes, showing significant improvement following tai chi intervention (MD: 0.88; 95% CI: 0.27 to 1.49; *p* = 0.005; *I*^2^ = 82%). Three studies reported MoCA results, indicating a substantial improvement compared to the control group (MD: 2.13; 95% CI: 1.25 to 3.02; *p* < 0.00001), although with high heterogeneity (*I*^2^ = 90%, *p* < 0.00001). The heterogeneity may stem from differences in study quality and design, suggesting the need for further subgroup analyses, such as comparing different intervention types (e.g., communication technology-based tai chi vs. traditional tai chi). Sensitivity analysis revealed no significant changes when individual studies were excluded, affirming the robustness of the results across all outcomes.

**Figure 5:**
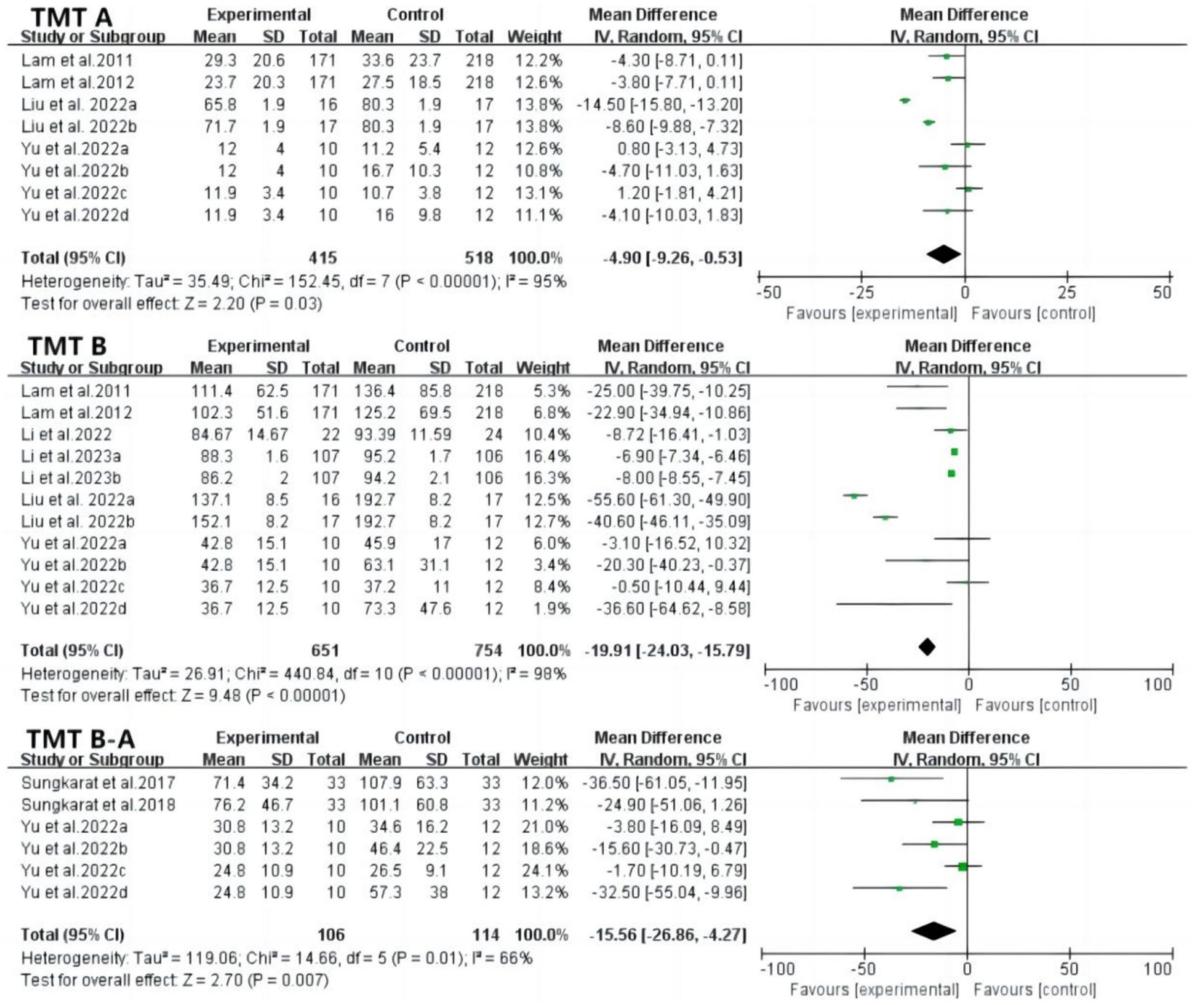
Forest plot of TMT A, TMT B and TMT B-A after tai chi intervention. TMT-A, trail making test A; TMT-B, trail making test B; TMT B-A, trail making test B-A.

Trail Making Test A (TMT A) scores effectively assessed visuo-spatial ability, showing significantly higher scores in the tai chi group compared to controls (MD: −4.90; 95% CI: −9.26 to −0.53; *p* = 0.03; *I*^2^ = 95%). Heterogeneity was notably reduced by excluding the study by Liu et al. (*p* = 0.11; *I*^2^ = 44%). Regarding executive function, six studies reported TMT B results (MD: −19.91; 95% CI: −24.03 to −15.79; *p* < 0.00001; *I*^2^ = 95%), and three studies reported TMT B-A results (MD: −15.56; 95% CI: −26.86 to −4.27; *p* = 0.007; *I*^2^ = 66%). Sensitivity analyses confirmed the robustness of these findings (Figure 5).

**Figure 6:**
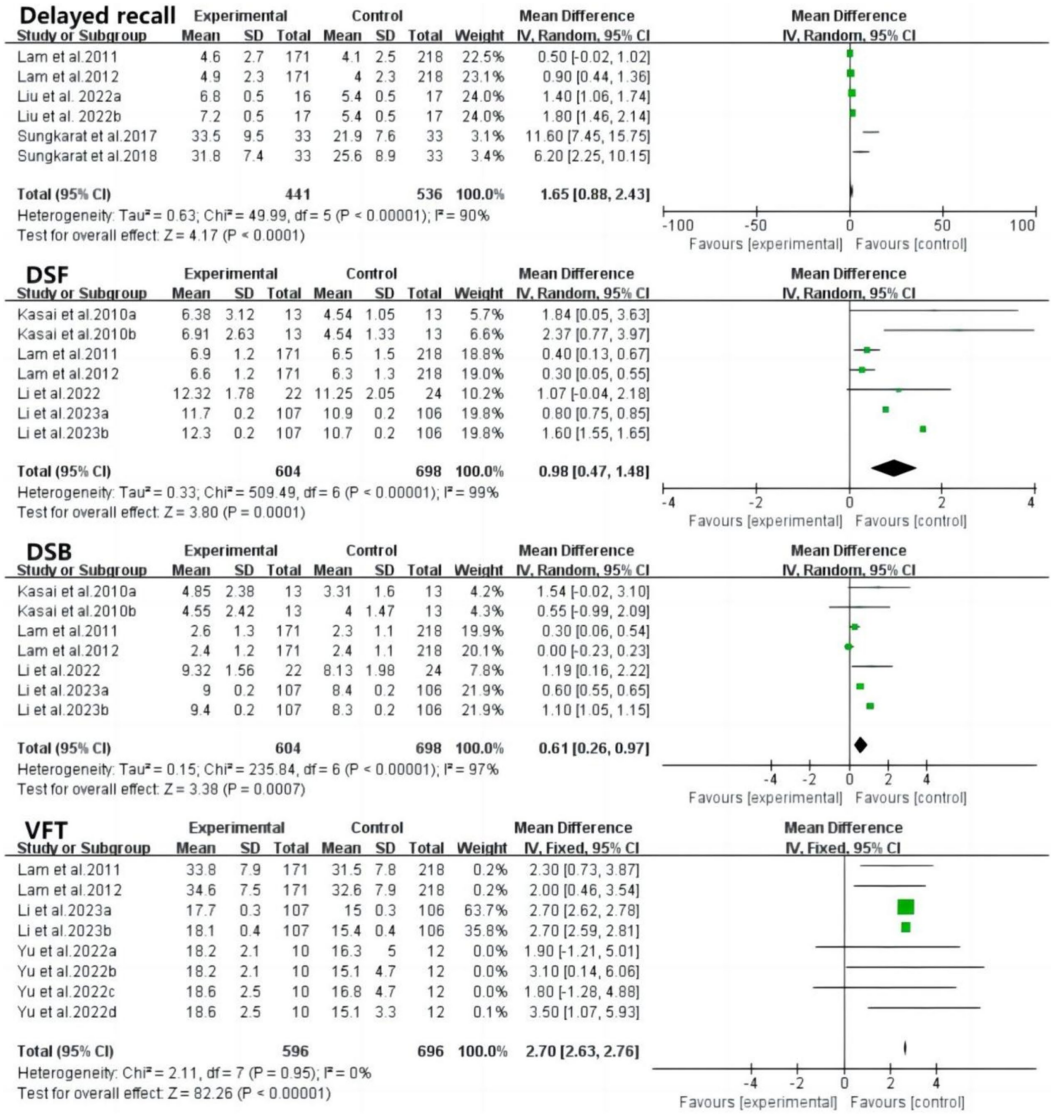
Forest plot of delayed recall, DSF, DSB, VFT after tai chi intervention. DSF, digital span forward; DSB, digital span backward; VFT, verbal fluency test.

Delayed recall, digital span forward, and digital span backward tests assessed memory function (Figure 6). Delayed recall scores were significantly higher after tai chi intervention (MD: 1.65; 95% CI: 0.88 to 2.43; *p* < 0.0001; *I*^2^ = 90%). Digital span forward and backward tests assessed working memory storage capacity, with both showing significant improvements (forward: MD: 0.98; 95% CI: 0.47 to 1.48; *p* = 0.0001; *I*^2^ = 99%; backward: MD: 0.61; 95% CI: 0.26 to 0.97; *p* = 0.0007; *I*^2^ = 97%). High heterogeneity suggests the need for further subgroup analyses to explore sources of variation. The verbal fluency test, assessing language function, showed significantly higher scores in the tai chi group, with no heterogeneity observed (MD: 2.70; 95% CI: 2.63 to 2.76; *p* < 0.00001; *I*^2^ = 0%).

### 3.4 Physical function

**Figure 7:**
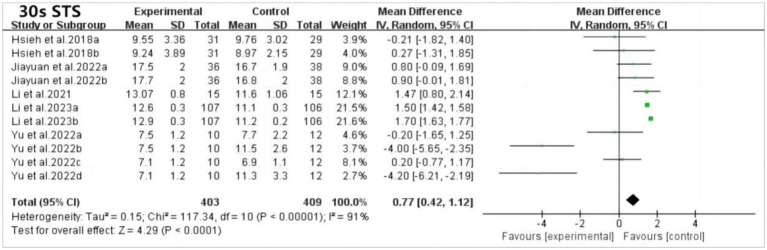
Forest plot of 30 s STS after tai chi intervention. 30 s-STS, 30 s sit to stand test.

The 30-s sit-to-stand test is a validated measure of lower limb muscle strength in older adults. Five studies examined the test, revealing significant improvements in performance among patients with mild cognitive impairment (MCI) compared to controls (MD: 0.77; 95% CI: 0.42 to 1.12; *p* < 0.0001). However, high heterogeneity was observed (*p* < 0.00001; *I*^2^ = 91%), likely due to variations in study characteristics. Sensitivity analysis showed no significant changes, highlighting the need for further subgroup analysis (Figure 7).

**Figure 8:**
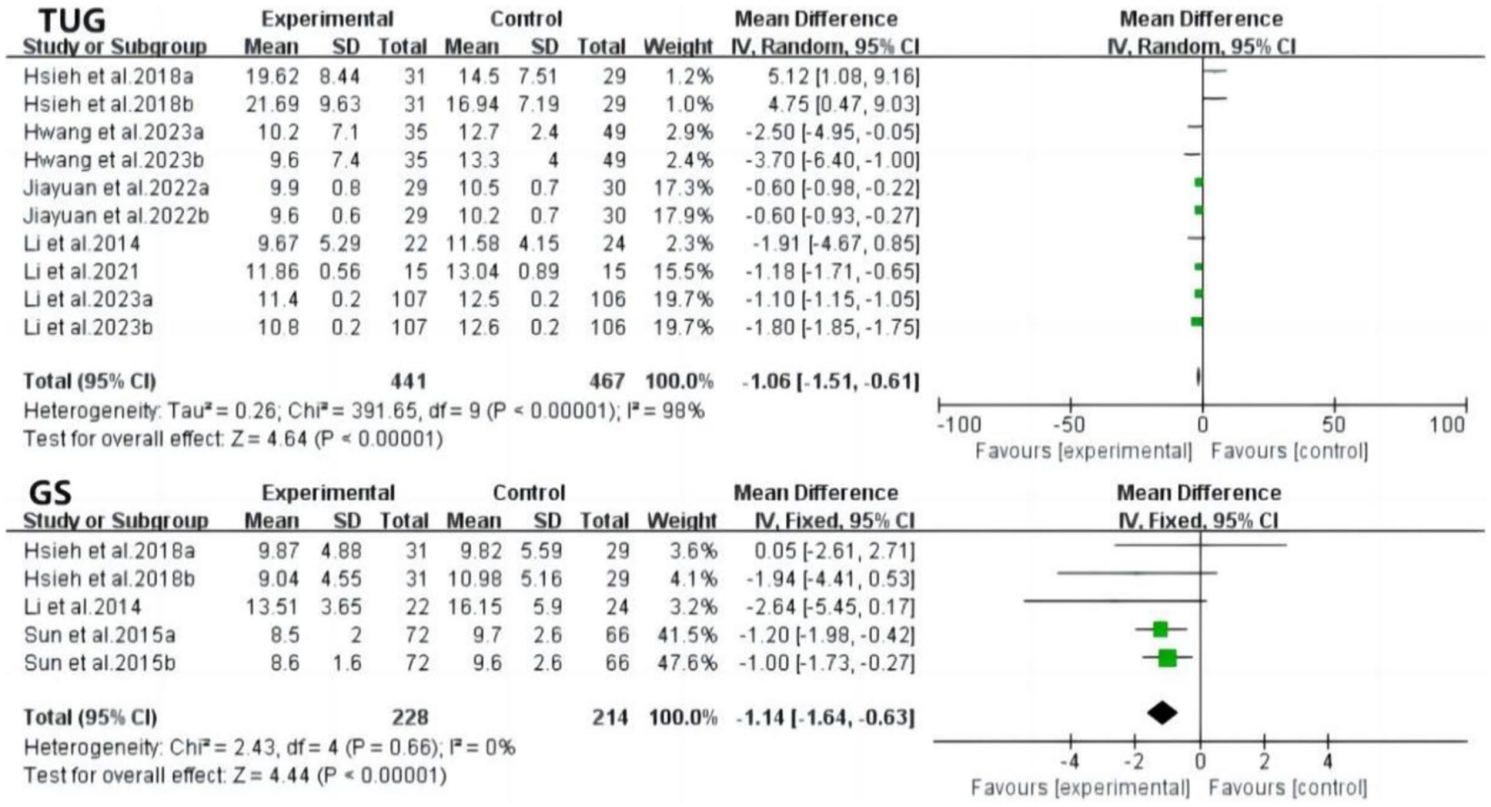
Forest plot of TUG and GS after tai chi intervention. TUG, timed up to go; GS, gait speed.

The Timed Up and Go (TUG) test evaluates functional walking ability in older adult individuals. The results demonstrated that tai chi training significantly improved TUG performance in patients with MCI compared to controls (MD: −1.06; 95% CI: −1.51 to −0.61; *p* < 0.00001; *I*^2^ = 98%). Similarly, gait speed, another measure of walking ability, showed significant improvement in the tai chi group (MD: −1.14; 95% CI: −1.64 to −0.63; *p* < 0.0001), with no heterogeneity (Figure 8).

**Figure 9:**
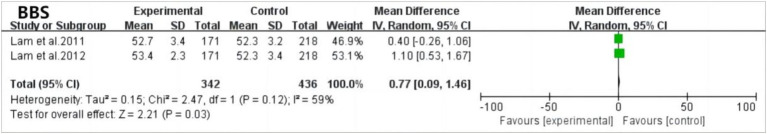
Forest plot of BBS after tai chi intervention. BBS, berg balance scale.

The Berg Balance Scale assesses dynamic and static balance in sitting and standing positions. After tai chi training, the experimental group showed significantly better Berg Balance Scale scores than the control group (MD: 0.77; 95% CI: 0.09 to 1.46; *p* = 0.03) (Figure 9).

### 3.5 Subgroup analysis

Subgroup analysis categorized studies by type of tai chi intervention, including traditional tai chi and ICT-based interventions (videoconferencing, video games, and virtual reality). Results indicated that ICT-based tai chi interventions showed significant improvements in all cognitive function indicators, while traditional tai chi also yielded significant results, except for the backward digit span test. This suggests that ICT-based interventions may outperform traditional tai chi for cognitive function. However, the limited number of ICT-based studies warrants further research. In terms of physical function, ICT-based tai chi significantly outperformed traditional tai chi in the 30-s sit-to-stand test and TUG scores, suggesting it may be more effective in improving physical function in MCI patients (Table 3).

**Table 3:** Subgroup comparison results of traditional and ICT-based tai chi.

Indicators	No of studies	Effect model	MD/SMD (95% Cl)	p
ICTTC				
Cognitive function				
MoCA	2	RE	1.38 [0.62, 2.14]	0.0004**
TMT-B	2	RE	−40.06 [−57.07, −23.05]	<0.00001**
DSF	2	RE	1.44 [0.73, 2.16]	<0.0001**
DSB	2	RE	1.64 [0.84, 2.44]	<0.0001**
Physical function				
30s STS	2	RE	0.71 [−0.40, 1.83]	0.21
TUG	2	RE	2.56 [−2.39, 7.51]	0.31
TC				
Cognitive function				
MoCA	1	RE	4.20 [1.59, 6.81]	0.002**
TMT-B	4	RE	−13.98 [−22.45, −5.52]	0.001**
DSF	3	RE	0.26 [−0.12, 0.64]	0.17
DSB	3	RE	0.37 [0.19, 0.55]	<0.0001**
Physical function				
30s STS	2	RE	−0.91 [−2.40, 0.57]	0.23
TUG	2	RE	−0.62 [−0.95, −0.29]	0.0002**

ICTTC, information and communication technologies-based tai chi; TC, tai chi; RE, random effect; MoCA, Montreal Cognitive Assessment; TMT-B, trail making test B; DSF, digital span forward; DSB, digital span backward; 30s-STS, 30s sit to stand test; TUG, timed up to go. *p < 0.1; **p < 0.01.

## 4 Discussion

This meta-analysis demonstrates the efficacy of tai chi interventions in improving both cognitive and physical functions in patients with MCI. In terms of cognitive function, significant improvements were observed across various tests, including Montreal Cognitive Assessment, Mini-Mental State Examination, Digital Span Forward, Digital Span Backward, Trail Making Test A, Trail Making Test B, Trail Making Test B-A, Verbal Fluency Test and Delayed Recall. These improvements reflect enhanced performance in key cognitive domains, such as visuospatial ability, executive function, memory, and language. These findings align with the results of previous meta-analyses (4–6, 21–24). Regarding physical function, participants in the tai chi group showed significant improvements in the 30-s sit-to-stand test, gait speed, and the Berg Balance Scale, indicating enhanced lower limb muscle strength, mobility, and balance in patients with MCI. Cognitive training through tai chi has been shown to improve cognitive function (25, 26). Compared with aerobic exercise, tai chi involves a greater number of specific movements performed in sequence, requiring participants to maintain focus, memorize, and repeatedly train, which enhances executive function and memory (23). The semi-squatting posture in tai chi increases the load on the lower limbs, inducing greater joint compression, while controlling limb movement and maintaining specific positions. This activates the tensor reflex, stimulating joint proprioceptors and enhancing the neuromuscular system’s responsiveness, enabling patients with mild cognitive impairment to react swiftly and accurately to environmental risks (27). Overall, tai chi improves physical function and enhances patients’ quality of life. The observed heterogeneity may be due to variations in study quality and design, suggesting that further subgroup analyses are needed to explore its sources, such as comparing different intervention types (e.g., ICT-based tai chi vs. traditional tai chi). Sensitivity analysis, after excluding studies one by one, showed no significant changes, confirming the robustness of the findings across all outcomes.

Regarding physical function, ICT interventions offer greater benefits than traditional approaches. Previous reviews have not examined the impact of ICT interventions on physical function, likely because most ICT interventions combine cognitive and exercise training, making it difficult to analyze due to the variety of intervention types. This study focused on a specific program, ICT-based tai chi, with strict inclusion criteria that excluded non-tai chi interventions. Tai chi itself serves as both a cognitive training and aerobic exercise process. Several studies (8, 16, 28) have integrated cognitive training with tai chi, and while findings suggest that ICT-based tai chi outperforms traditional tai chi, it complicates understanding the source of the intervention’s effect. The ICT modalities in this research included virtual reality, exergames, and videoconferencing. Virtual reality and exergames increased patient interest and exercise enjoyment, while videoconferencing was less engaging and had lower patient adherence. In three studies using videoconferencing, one study (29) found no significant improvement in physical function, while two reported positive outcomes (8, 16). Further analysis suggests that supervision and evaluation methods played a crucial role in these results. The two studies with better outcomes provided more detailed supervision and assessed factors such as exercise safety, enjoyment, intensity appropriateness, brain health benefits, and overall satisfaction—criteria that should exceed 70% satisfaction. This highlights the potential of ICT not only for treatment but also for monitoring and evaluating therapeutic effects (30). Various ICT-based monitoring and evaluation tools, such as fitness trackers, have been developed and can promote patient self-management, helping delay declines in cognitive and physical function (31, 32). As technology advances and costs decrease, more patients could benefit from refined ICT-based rehabilitation programs.

Subgroup analysis results indicate that ICT-based tai chi is more effective in enhancing cognitive and physical performance compared to traditional teaching and exercise methods. The study concludes that information and communication technologies (ICT) can activate and improve the cognitive functions of patients with mild cognitive impairment by fostering increased interaction. In virtual environments such as virtual reality (VR) and exergames, patients must remain alert to feedback on situational changes and adjust their posture in response to commands. ICT-based tai chi interventions provide an immersive experience that enhances motivation and interest in the activity. VR, in particular, can integrate a complex array of stimuli—such as vivid imagery and realistic sounds—that help users feel physically present in an engaging environment (9), thus enhancing enjoyment. These virtual interactions simulate real physical, visual, and auditory sensations, creating a sense of immersion that alleviates anxiety and reduces behavioral symptoms (9). This process activates the brain’s sensorimotor integration function and stimulates attention-related brain networks (33). Virtual environments provided by computers or VR devices improve attention in patients with mild cognitive impairment, stimulating brain areas responsible for cognitive and neuromuscular control, which strengthens brain circuits (34). While fewer studies focus on remote instruction through videoconferencing, evidence supports its effectiveness in cognitive rehabilitation (8, 29). Multiple studies have reinforced this conclusion (35–37), suggesting that emerging technologies can achieve therapeutic effects comparable to or even surpassing those of traditional treatments. However, it is important to note that prolonged use of such devices may induce nausea and motion sickness, particularly among older adults susceptible to these effects. Future research should explore ways to mitigate these potential negative outcomes. Moreover, researchers and practitioners are encouraged to use the RE-AIM framework to design dissemination, evaluate interventions, and set up the relevant dimensions of factors with a beginning-end in mind (38–40). Such judicious application of RE-AIM will facilitate the replicability and generalizability of program interventions for optimal public health impact.

In conclusion, the study confirmed the effectiveness of tai chi intervention, especially ICT-based tai chi interventions, on the cognitive and physical functioning aspects of patients with mild cognitive impairment, and these findings provide valuable guidance programs for clinical practice and public health with older adult patients. However, there are some limitations exist. First, the variability in ICT-based tai chi interventions across studies necessitates further research into how different VR techniques and ICT modalities impact outcomes, along with the development of detailed intervention protocols to maximize therapeutic efficacy. Second, the combination of traditional and ICT-based tai chi complicates the isolation of the specific effects of ICT-based tai chi, which may result in conclusions that require cautious application. Third, as tai chi is both a physical and mental exercise, the instructor’s teaching philosophy, level of expertise, and methodology can significantly influence intervention outcomes. Future research should explore the integration of traditional and ICT-based tai chi to leverage the strengths of both, creating an effective tai chi rehabilitation program. In addition, subsequent studies need to learn more about the framework for the use of RE-AIM in a variety of contexts, assessing the relevant dimensions of the intervention and setting factors to help plan the replicability and generalizability of the intervention for optimal public health impact.

## 5 Conclusion

Tai chi interventions have positive improvement benefits for patients with mild cognitive impairment, and tai chi integrated with communication-based technologies in particular further improves the effectiveness of their cognitive and physical functioning. Better treatment protocols could improve the health of an increasing number of patients with mild cognitive impairment, which provides a scientific basis for the development of intervention programs and clinical practice for older adults.

## Data Availability

The original contributions presented in the study are included in the article/Supplementary material, further inquiries can be directed to the corresponding author.
