# Supplementary material for: Effects of tai chi based on information and communication technology for patients with mild cognitive impairment on cognitive and physical function: a systematic review and meta-analysis
Source: Front Public Health. 2025 Jan 7;12:1495645. doi: 10.3389/fpubh.2024.1495645 (PMC11748305; doi:10.3389/fpubh.2024.1495645)
Supplement: Supplementary file 1 [file Data_Sheet_1.docx]

**search strategy for Pubmed and Cochrane**

| Search | Search Terms |
| --- | --- |
| #1 | (Tai-ji or Tai Chi or Tai Ji Quan or Taiji or Taijiquan or T'ai Chi or Tai Chi Chuan) |
| #2 | (mild cognitive impairment or patients with mild cognitive impairment or mild cognition impairment or mild cognitive impairment identification or early mild cognitive impairment or lightly cognitive impediment or mild cognitive disorders or mild cognitive or Cognitive Dysfunctions or Cognitive Impairments or Cognitive Impairment or Cognitive Disorder or Cognitive Disorders or Mild Cognitive Impairment or Mild Cognitive Impairments or Cognitive Decline or Cognitive Declines or Mental Deterioration or Mental Deteriorations) |
| #3 | (Gait or Balance or 6MWT or Six minutes´ walk test or ABC or Activities-specific Balance or PH or Physical Health or SPPB or The Short Physical Performance Battery or TUG or Time up and go Test or Physical function or Cognitions or Cognitive Function or Cognitive Functions or Motor function ) |
| #4 | 1 AND 2 AND 3 |

**search strategy for Web of Science**

| Search | Search Terms |
| --- | --- |
| #1 | TS=(Tai-ji or Tai Chi or Tai Ji Quan or Taiji or Taijiquan or T'ai Chi or Tai Chi Chuan) |
| #2 | TS=(mild cognitive impairment or patients with mild cognitive impairment or mild cognition impairment or mild cognitive impairment identification or early mild cognitive impairment or lightly cognitive impediment or mild cognitive disorders or mild cognitive or Cognitive Dysfunctions or Cognitive Impairments or Cognitive Impairment or Cognitive Disorder or Cognitive Disorders or Mild Cognitive Impairment or Mild Cognitive Impairments or Cognitive Decline or Cognitive Declines or Mental Deterioration or Mental Deteriorations) |
| #3 | TS=(Gait or Balance or 6MWT or Six minutes´ walk test or ABC or Activities-specific Balance or PH or Physical Health or SPPB or The Short Physical Performance Battery or TUG or Time up and go Test or Physical function or Cognitions or Cognitive Function or Cognitive Functions or Motor function ) |
| #4 | 1 AND 2 AND 3 |

**search strategy for Elseiver**

| Search | Search Terms |
| --- | --- |
| #1 | (Tai chi or Tai chi chuan) |
| #2 | (mild cognitive impairment) |
| #3 | (Cognitive Functions or Motor function ) |
| #4 | 1 AND 2 AND 3 |
